# Integrating microbiome insights into cervical cancer

**DOI:** 10.3389/fmed.2026.1766052

**Published:** 2026-03-02

**Authors:** Jhommara Bautista, Paula Cortiñas Sardi, Iván Paguay-Caisabanda, Kelly Gancino-Guevara, Andrés López-Cortés

**Affiliations:** 1 Cancer Research Group (CRG), Faculty of Medicine, Universidad de Las Américas, Quito, Ecuador; 2 Department of Gynecology, Faculty of Medicine, Universidad Central de Venezuela, Escuela Luis Razetti, Caracas, Venezuela

**Keywords:** cervical cancer, cervicovaginal microbiome, community state type IV, HPV, HPV clearance, immune modulation, *Lactobacillus crispatus*, microbiome-based biomarkers

## Abstract

Cervical cancer is largely preventable, yet it continues to cause substantial morbidity and mortality worldwide, particularly in regions with limited access to human papillomavirus (HPV) vaccination and screening. Persistent infection with high-risk HPV genotypes, especially HPV-16 and HPV-18, represents the primary initiating event in cervical carcinogenesis. However, viral infection alone does not fully explain why only a subset of infected individuals develop high-grade lesions or invasive disease. Recent longitudinal and mechanistic studies indicate that the cervicovaginal microbiome plays an important modulatory role by influencing epithelial barrier integrity, local immune responses, and inflammatory homeostasis. This review synthesizes current evidence from multi-omics and translational studies linking cervicovaginal microbial composition and function to HPV persistence, cervical intraepithelial neoplasia (CIN), and cervical cancer progression. Communities dominated by *Lactobacillus crispatus* are frequently associated with antiviral conditions and mucosal stability, whereas anaerobe-enriched microbial profiles, commonly referred to as community state type IV (CST IV), are associated with chronic inflammation, metabolic dysregulation, and increased lesion severity. Microbial metabolites and inflammatory mediators may interact with HPV oncogene activity and host epigenetic regulation, supporting a microbiome-metabolome-epigenome axis in cervical carcinogenesis. The review also discusses emerging clinical implications, including microbiome-based biomarkers and microbiota-targeted interventions. While early studies suggest potential benefits of probiotics and postbiotics for HPV clearance and immune modulation, current evidence remains limited. Methodological heterogeneity, low-biomass sampling, and population variability continue to restrict causal inference and clinical translation. Addressing these challenges will be essential for integrating microbiome-informed strategies into cervical cancer prevention and management.

## Introduction

Cervical cancer remains a major global public health challenge, with approximately 640,000 new cases and 350,000 deaths reported worldwide in 2022, ranking as the fourth most common malignancy among women. The disease burden is disproportionately concentrated in low Human Development Index (HDI) regions, where limited access to human papillomavirus (HPV) vaccination, screening, and early treatment continues to drive high incidence and mortality rates. Epidemiological projections based on Bayesian age-period-cohort models estimate that cervical cancer incidence may increase by more than 50% by 2050 if current prevention strategies are not substantially strengthened. Persistent infection with high-risk human papillomavirus (hrHPV), particularly HPV-16 and HPV-18, represents the central initiating event in cervical carcinogenesis, primarily through the oncogenic activities of the viral E6 and E7 proteins, which inactivate p53 and pRb, respectively, thereby promoting genomic instability and immune evasion. Population-level data demonstrate marked reductions in cervical intraepithelial neoplasia grade 3 (CIN3) and invasive cancer among women vaccinated prior to sexual debut, reinforcing the largely preventable nature of this malignancy. Nevertheless, several cofactors, including tobacco exposure, long-term hormonal contraception, HIV-associated immunosuppression, and alterations in the cervicovaginal microenvironment, modulate the progression from HPV infection to invasive disease (1–3).

Although vaccination has proven highly effective, global HPV vaccine coverage remains alarmingly low. A recent systematic review encompassing 148 national immunization programs reported that, as of 2023, only 27% of girls worldwide had received at least one vaccine dose, and merely 20% had completed the full vaccination schedule, well below the 90% coverage target established by the World Health Organization for 2030. Moreover, the occurrence of breakthrough infections and persistent hrHPV carriage in vaccinated and unvaccinated populations underscores the need to identify additional biological determinants of viral persistence and malignant transformation. In this context, the cervicovaginal microbiome has emerged as a clinically relevant modulator of cervical cancer risk. Recent multi-omics and longitudinal cohort studies position the cervicovaginal microbiome as a functional axis interacting with HPV exposure and host genetics, influencing epithelial barrier integrity, innate immune activation, inflammatory tone, and local metabolite production (4–6). The magnitude and direction of these interactions differ across ethnic groups, hormonal states, and environmental settings. Similar microbial configurations may therefore exert distinct biological effects depending on host susceptibility and baseline immune tone. Framing microbiome-host interactions as gradients of risk rather than fixed causal pathways more accurately reflects current evidence (7, 8). Incorporating microbiome-derived biomarkers into cervical cancer prevention frameworks therefore represents a promising opportunity to enhance risk stratification and public health impact Importantly, while early studies were largely cross-sectional, recent longitudinal cohorts indicate that baseline cervicovaginal microbiome states precede and predict HPV persistence and disease progression, supporting temporal precedence as a prerequisite for causal inference (9–11).

Beyond static compositional changes, temporal regulation of host-microbiota interactions has gained attention as an additional layer of biological complexity in cervical carcinogenesis. Circadian misalignment, driven by irregular light exposure, sleep disruption, or altered feeding rhythms, has been shown to perturb microbial oscillatory behavior, leading to dysbiosis, immune desynchronization, and sustained low-grade inflammation. Approximately 10–15% of microbial taxa, including *Lactobacillus* and *Bacteroides* species, exhibit time-of-day dependent fluctuations in abundance and transcriptional activity, with downstream effects on metabolite production such as short-chain fatty acids and bile acids. Disruption of these rhythmic outputs compromises epithelial defense mechanisms and amplifies pro-inflammatory signaling pathways, processes increasingly recognized as contributors to HPV persistence and neoplastic progression within the cervicovaginal niche (12, 13).

Parallel advances in cancer biology further highlight the bidirectional interplay between microbial ecosystems and tumor progression (14). Tumor-derived extracellular vesicles have been shown to condition local and distant microenvironments by altering vascular permeability, immune cell recruitment, and stromal remodeling in an organ-specific manner. Importantly, several of these processes appear to be temporally regulated and synchronized with host circadian and immune rhythms. Although most evidence derives from solid tumors outside the cervix, these findings provide a mechanistic framework to explore how immune desynchronization and microbial dysbiosis may cooperate to facilitate HPV-driven cervical carcinogenesis (15).

High-resolution multi-omics studies increasingly portray the microbiome as an active regulator of tumor biology rather than a passive bystander. In the gastrointestinal tract, commensal taxa such as *Akkermansia muciniphila*, *Faecalibacterium prausnitzii*, and selected *Bifidobacterium* species enhance dendritic cell priming and CD8^+^ T-cell activation, correlating with improved responses to immune checkpoint inhibitors. Conversely, antibiotic-induced dysbiosis impairs therapeutic efficacy and promotes immune dysfunction. Microbial metabolites, including indole derivatives and short-chain fatty acids, modulate oxidative stress responses and influence sensitivity to chemotherapy in preclinical models. Beyond the gut, viable bacteria and fungi have been detected within tumor tissues across multiple cancer types, where they reshape metabolic fluxes, immune surveillance, and therapeutic response. Early-phase clinical trials of fecal microbiota transplantation (FMT) further support the translational relevance of microbiome modulation, although heterogeneity in outcomes highlights the need for precision-based approaches integrating microbial function, host immunity, and genetic context (16–18).

Building on this broader multi-omics framework, recent large-scale metagenomic and multi-center immunotherapy studies further reinforce the functional relevance of the microbiome in cancer progression and treatment response. In particular, a conserved gut microbial signature enriched in *Akkermansia*, *Faecalibacterium*, and *Bifidobacterium* species has been associated with improved outcomes to PD-1 and combined PD-1/CTLA-4 blockade across multiple solid tumor types, including melanoma and lung cancer (19). Antibiotic-induced disruption of these communities correlates with impaired antitumor immunity and therapeutic resistance. Complementing these observations, intratumoral microbiota have emerged as active modulators of tumor metabolism, immune surveillance, and drug response, with bacteria detected intracellularly within cancer cells and immune compartments. Although preclinical FMT studies demonstrate reproducible restoration of immunotherapy efficacy, early clinical trials and meta-analyses reveal substantial inter-patient heterogeneity and unresolved safety concerns, underscoring the current translational limitations of microbiome-based interventions in oncology (20, 21).

Within this broader oncological landscape, the cervicovaginal tract constitutes the primary ecological niche for HPV acquisition, persistence, and malignant progression. In many individuals, *Lactobacillus crispatus*-dominant communities are commonly associated with a low-pH, lactic acid-rich environment that restricts viral replication and supports mucosal immune homeostasis; however, stable and asymptomatic cervicovaginal ecosystems can also occur under alternative microbial configurations depending on host factors and functional outputs rather than taxonomic dominance alone. In contrast, shifts toward anaerobe-dominated community state types, characterized by taxa such as *Gardnerella*, *Prevotella*, and *Sneathia*, are consistently associated with increased risk of persistent hrHPV infection, high-grade cervical lesions, and invasive cancer. Metagenomic and metabolomic studies supporting an emerging model in which dysbiosis-associated metabolites may influence epithelial integrity, interferon signaling, and epigenetic regulation, potentially intersecting with HPV oncogene–driven pathways. These insights have stimulated interest in microbiome-informed diagnostics and early interventions aimed at restoring microbial equilibrium and enhancing viral clearance (22–24).

Longitudinal and integrative studies further demonstrate that baseline microbiome configurations predict subsequent HPV clearance or persistence over time, providing evidence for temporal directionality rather than static association. Women harboring *Lactobacillus*-dominated communities exhibit enhanced antiviral immunity and higher rates of viral clearance, whereas anaerobe-enriched dysbiotic states are consistently associated with immune dysfunction, sustained inflammation, and increased risk of lesion progression. Metagenomic and metatranscriptomic analyses reveal that HPV infection itself can reshape microbial gene expression and metabolic output, reinforcing a bidirectional feedback loop between viral oncogenesis and microbial dysbiosis. Supporting evidence from other mucosal sites, including the oral cavity, further suggests that HPV-associated microbial alterations may represent a conserved host–microbe interaction pattern across epithelial tissues, providing comparative insight into HPV-driven carcinogenesis beyond the cervix (25–27). However, whether these conserved patterns reflect shared causal mechanisms or parallel dysbiotic responses remains to be established.

**Figure 1:**
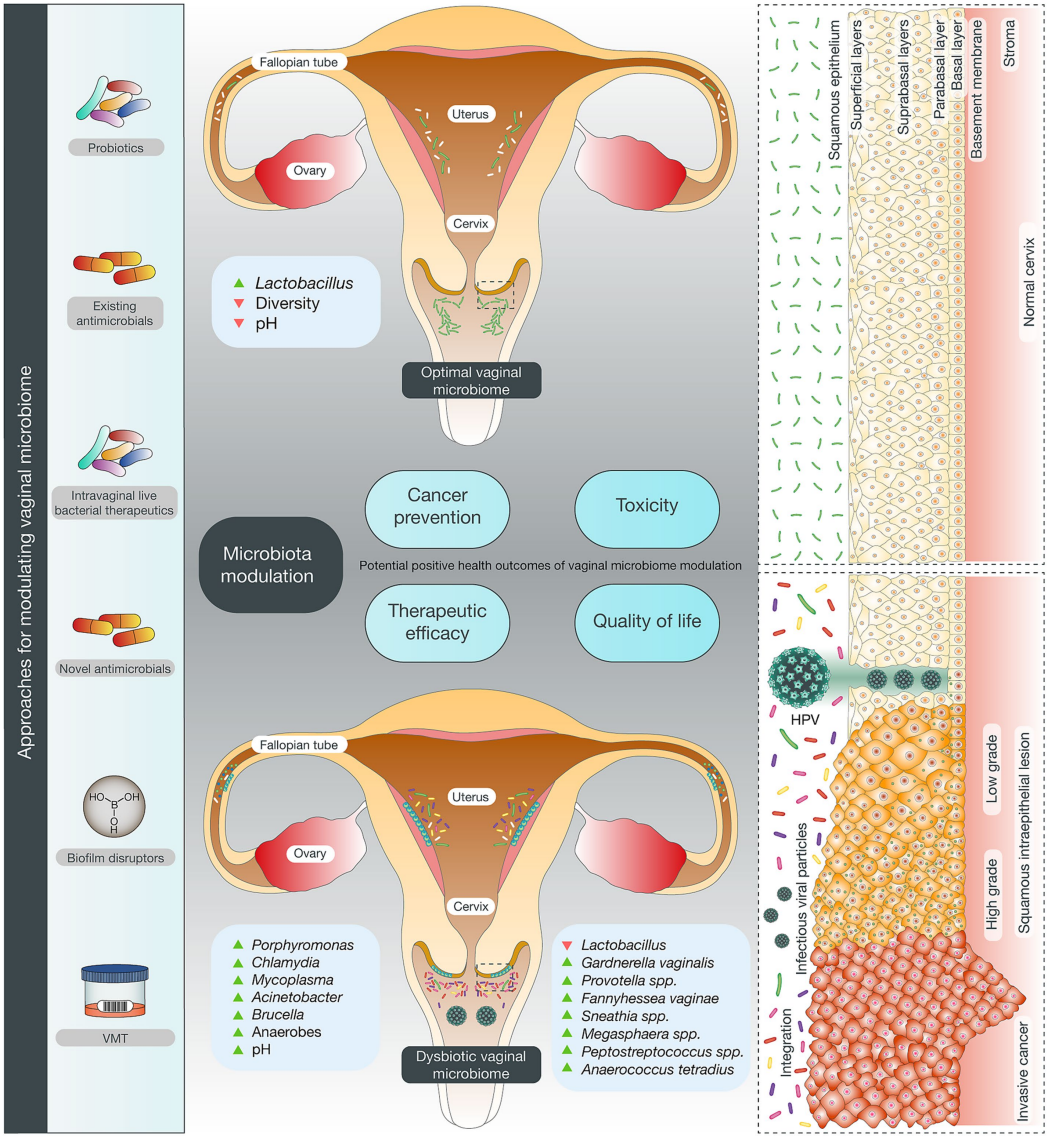
The figure depicts the interconnected processes linking high-risk HPV infection, cervical epithelial transformation, and microbiota-driven modulation of disease progression. In healthy reproductive-age women, the cervix and vagina harbor *Lactobacillus*-dominant microbial communities that generate an acidic microenvironment, sustain epithelial barrier integrity, and reinforce innate antiviral immunity. These features help restrict pathogen overgrowth and often limit HPV infections to a transient phase. Persistent HPV infection and progression to high-grade lesions or invasive cancer coincide with profound cervicovaginal dysbiosis, marked by reduced *Lactobacillus* abundance and expansion of pro-inflammatory anaerobes such as *Sneathia*, *Gardnerella*, *Fannyhessea vaginae*, *Prevotella*, *Megasphaera*, *Anaerococcus*, and *Peptostreptococcus*. The resulting elevation in vaginal pH, erosion of epithelial defenses, and heightened inflammatory signaling collectively promote viral persistence, immune evasion, and neoplastic remodeling. A growing set of microbiome-informed interventions aims to restore ecological balance and support HPV clearance. These approaches include targeted antimicrobials, biofilm-disrupting agents, next-generation probiotics, intravaginal *Lactobacillus* formulations, prebiotic substrates, vaginal microbiota transplantation, recombinant bacterial vaccines, microbial metabolite modulation, and engineered microbial immunotherapies. Such strategies hold potential to enhance treatment responsiveness, reduce toxicity, and improve outcomes in HPV-associated cervical disease.

Nevertheless, establishing causality in human microbiome research remains inherently challenging, given the dynamic and context-dependent nature of microbial ecosystems. Despite rapid advances in cervicovaginal microbiome research, several limitations constrain current interpretation and clinical translation. Most available studies are cross-sectional, limiting causal inference between microbial dysbiosis and cervical carcinogenesis. Methodological heterogeneity, including variability in sampling procedures, sequencing strategies (16S rRNA versus shotgun metagenomics), bioinformatic pipelines, and cohort composition, complicates cross-study comparisons. The low-biomass nature of cervicovaginal samples further increases susceptibility to contamination and technical bias, particularly in tumor-adjacent tissues. In addition, populations from low-resource settings, where cervical cancer burden is highest, remain underrepresented in existing datasets (28–30). This review therefore aims to critically synthesize mechanistic, multi-omics, and translational evidence while explicitly acknowledging current gaps, with the goal of identifying robust biological signals and guiding future microbiome-informed strategies for cervical cancer prevention and management. Accordingly, while accumulating longitudinal, mechanistic, and interventional evidence supports a contributory causal role of the cervicovaginal microbiome, definitive causality will require large-scale prospective studies with repeated sampling and controlled microbiome-targeted interventions (Figure 1).

## Characterization of the cervicovaginal microbiome in cervical cancer

### Composition of the healthy versus dysbiotic vaginal microbiome

Large-scale studies have reinforced the utility of the community state type (CST) framework initially established through 16S rRNA surveys. In healthy, reproductive-age women, over 70% of samples cluster into CST I–III, dominated, respectively, by *Lactobacillus crispatus, Lactobacillus gasseri*, and *Lactobacillus iners.* These taxa maintain a low vaginal pH (<4.5) via lactic acid, hydrogen peroxide, and bacteriocin production, while strengthening epithelial barrier function and activating type I interferon signaling. Metabolomic analyses indicate that *L. crispatus* uniquely produces *ω*-hydroxylated fatty acids with antimicrobial properties that restrict anaerobic overgrowth. By contrast, *L. iners*, characterized by inerolysin expression and an incomplete D-lactate pathway, are associated with transitional CST profiles prone to instability under hormonal or inflammatory stress (31–33). The CST framework reflects compositional clustering rather than intrinsic health or disease states. Although CST-IV is operationally defined as a Lactobacillus-depleted, higher-diversity community, it comprises biologically distinct ecological subtypes with divergent inflammatory and metabolic features. In some populations, *Lactobacillus*-depleted communities may represent stable, asymptomatic configurations. Epidemiological studies consistently demonstrate marked ethnic, geographic, and hormonal variation in CST distribution, indicating that microbial risk profiles are shaped by host and environmental context rather than universally defined by *Lactobacillus* dominance (34, 35). For this reason, associations between CST patterns and HPV outcomes should be interpreted relative to population-specific baselines and relevant confounders, including contraceptive use, pregnancy status, co-infections, smoking, and socioeconomic determinants, all of which can shift similar taxonomic profiles toward distinct functional or inflammatory states (36, 37). The same taxonomic profile may be linked to divergent inflammatory or antiviral responses in distinct demographic settings. Interaction strength is therefore context-dependent, and CST categories should be understood as ecological descriptors rather than biologically uniform states (38).

Beyond taxonomic composition, cervicovaginal communities function as structured ecological networks in which stability emerges from interspecies competition, metabolic cooperation, and spatial organization. *Lactobacillus*-dominated states are maintained not only through host immune support but also via competitive exclusion mechanisms, including rapid glycogen utilization, acidification, and bacteriocin-like activity that suppress facultative and obligate anaerobes. By contrast, higher-diversity communities frequently exhibit metabolic cross-feeding, whereby primary degraders release substrates such as amino acids or mucin-derived sugars that secondary fermenters exploit. Such interactions can reinforce community persistence and alter local pH independently of direct host modulation (39, 40).

CST-IV communities are typically characterized by diminished Lactobacillus abundance, increased α-diversity, and enrichment of anaerobic taxa including *Gardnerella vaginalis*, *Atopobium vaginae*, *Prevotella*, *Sneathia*, *Megasphaera*, and *Mobiluncus*. These organisms produce polyamines such as putrescine and cadaverine, which elevate vaginal pH, compromise mucus integrity, and promote inflammatory signaling pathways involving interleukin-1β, tumor necrosis factor-α, and matrix metalloproteinases. Long-read metagenomic analyses have further revealed multi-kingdom dysbiosis, including enrichment of anelloviruses and *Candida* species in selected contexts (41–43). Despite fluctuations associated with menstrual cycling, infection, or antibiotic exposure, pregnancy and targeted probiotic interventions may facilitate re-establishment of *Lactobacillus*-dominated configurations, underscoring the plasticity of the cervicovaginal ecosystem. At the metabolite level, strain-specific functional outputs further distinguish Lactobacillus species. For example, *L. crispatus* produces linoleic acid–derived 10-hydroxy-12-octadecenoic acid, a biosurfactant with antimicrobial activity against pathogens such as *Chlamydia trachomatis* (32, 44).

### Dysbiosis and its association with high-risk HPV persistence

Longitudinal cohorts demonstrate that women with CST IV at baseline have a two- to four-fold greater risk of persistent high-risk human papillomavirus (hrHPV) infection. Experimental epithelial and *in vitro* models demonstrate that Gardnerella-dominated biofilms can impair STING-dependent interferon signaling and reduce E-cadherin integrity, thereby weakening epithelial defenses. Complementary shotgun metagenomic analyses have identified enrichment of microbial genes encoding L-serine dehydratase and polyamine biosynthetic enzymes in dysbiotic communities. These functional inferences suggest a potential contribution to host oncogenic polyamine flux, although *in vivo* validation in human cervical tissue remains limited (25, 26, 45). Biofilm architecture illustrates the central role of microbe-microbe interactions in sustaining dysbiosis. *Gardnerella vaginalis* frequently serves as a structural scaffold that enables adhesion of *Prevotella, Atopobium,* and *Sneathia* species within polymicrobial biofilms. Functional complementarity within these communities enhances ecological stability: mucin-degrading bacteria release simple carbohydrates and amino acids that support fermentative taxa, which subsequently generate short-chain fatty acids and polyamines capable of modifying pH and redox balance. This coordinated metabolic activity increases resistance to immune clearance and antimicrobial perturbation. Persistent dysbiosis therefore reflects community-level organization and metabolic cooperation rather than isolated virulence traits, providing a mechanistic framework for its association with HPV persistence and lesion progression (9, 46). Transitions toward *Lactobacillus crispatus*-dominant states often coincide with viral clearance; however, this association is not universal. Effective HPV control has also been documented in alternative microbial configurations, including selected non-*Lactobacillus* communities, depending on host immune competence and microbial functional outputs. Risk appears to correlate more consistently with functional signatures, including biofilm architecture, mucin turnover, polyamine flux, lactate stereochemistry, and interferon pathway modulation, than with community state type labels alone (47, 48). Machine learning models integrating microbial abundances such as *Lactobacillus, Prevotella,* and *Sneathia* with metabolite ratios, including lactic acid to putrescine, have achieved area under the curve values exceeding 0.85, outperforming genotype- and cytology-based predictors (49). Clinical implementation remains constrained by small probiotic intervention cohorts and limited multi-ethnic validation.

### Progressive microbiome shifts across precancerous lesions and invasive cancer

Nested case–control and longitudinal studies consistently show microbiome alterations paralleling progression from normal epithelium to CIN1/2, CIN3, and invasive squamous cell carcinoma (SCC). Early lesions often retain CST III (*L. iners*-dominant), whereas advanced disease is characterized by expansion of anaerobic taxa such as *Fusobacterium*, *Peptostreptococcus*, and tumor-associated *Streptococcus* species, alongside depletion of *Lactobacillus* (50, 51). Metabolomic shifts, including rising succinate, β-hydroxybutyrate, and polyamines, reinforce tumor-promoting hypoxia-inducible factor-1α (HIF-1α) signaling and immune suppression. Spatial transcriptomics further localizes *Sneathia* to epithelial zones with p16^INK4a overexpression and NF-κB activation, suggesting direct oncogenic signaling links (52, 53). Intratumoral analyses also detect intracellular *Pseudomonas* and *Enterobacter* capable of metabolizing cisplatin, potentially contributing to chemoresistance. Importantly, post-treatment CST IV-like microbiomes predict residual dysplasia and recurrence risk, whereas re-dominance of *Lactobacillus* post-LEEP strongly correlates with sustained hrHPV negativity. Consistent with these trajectories, intratumoral and even intracellular bacteria can drive EMT, remodel ECM, and bias immune surveillance, providing a plausible mechanistic bridge between CST-IV dysbiosis and invasion, persistence, and treatment resistance (54–56).

### Omics techniques and methodological challenges

While 16S rRNA sequencing remains cost-effective for population-level studies, its resolution is limited (e.g., collapsing *Gardnerella* clades) and it cannot capture viruses or fungi. Shotgun metagenomics provides strain-level resolution, functional pathway mapping, and multi-kingdom profiling but faces cervicovaginal-specific hurdles: host DNA contamination (>95% of reads), low biomass, and incomplete reference genomes. Recently optimized host-depletion methods (saponin, methyl-CpG capture, CRISPR-Cas) have improved microbial read yield up to 30-fold without compositional bias. Hybrid approaches combining shallow shotgun with long-read scaffolding now enable strain-resolved assemblies. Single-cell metagenomics and spatial meta-omics are emerging as tools for mapping host–microbe interaction networks *in situ*. However, standardized protocols for extraction, sequencing depth, and bioinformatics are needed to ensure reproducibility. Although CST assignment via 16S and VALENCIA classifiers is efficient for research, only shotgun metagenomics meets clinical-grade standards, exemplified by the CLIA/CAP/CLEP-approved Evvy platform, reporting >90% sensitivity and specificity in >7,000 samples, already applied in remote bacterial vaginosis management. Other multi-omic strategies, including metatranscriptomics, metabolomics, and single-cell analysis, remain promising but experimental, lacking validation for clinical practice (57–59).

## Molecular and immunological mechanisms involved in cervical carcinogenesis

### Inflammatory pathway activation

Inflammation is a key driver of cervical carcinogenesis, providing the mechanistic link between dysbiosis-derived microbial signals and dysregulated host immunity. Cervical epithelial cells express diverse pattern recognition receptors (PRRs), notably Toll-like receptors (TLRs), which detect microbial pathogen-associated molecular patterns (PAMPs) and initiate innate immune signaling (60). Under dysbiotic conditions, excessive activation of TLRs by bacterial PAMPs, particularly lipopolysaccharide (LPS) from Gram-negative species, triggers NF-κB signaling and downstream pro-inflammatory cascades, as shown in SiHa cervical carcinoma cells (61, 62). Overexpression of TLR4 in cervical tumor tissues further amplifies HIF-1α signaling and drives secretion of immunosuppressive cytokines, including IL-6 and TGF-β1, which facilitate immune evasion, proliferation, and autophagy in tumor-associated macrophages (62, 63). Parallel findings indicate that IL-1β potentiates NF-κB signaling and induces CCL-2 expression in HeLa cells (64), while high IL-1β levels in the tumor microenvironment correlate with aggressive phenotypes and angiogenesis (65). Sustained NF-κB activation thus represents a hallmark of chronic inflammation in cervical cancer, associated with poor prognosis and increased mortality (66–68).

### Immune modulation in the cervical transformation zone

Although macrophages, CD4^+^/CD8^+^ T cells, and immature dendritic cells are present within the transformation zone, where up to 90% of cervical tumors originate, an increasingly immunosuppressive milieu dominates during neoplastic progression (69, 70). High CXCL12 levels impede effector T cell infiltration while promoting regulatory T cell (FoxP3^+^) expansion, thereby reinforcing local suppression (71). In addition, tumor-derived HMGB1 alters plasmacytoid dendritic cell (pDC) function by suppressing IFN-α release and upregulating ICOSL, which enhances Treg proliferation and IL-10 secretion (72, 73). IL-6 plays a dual role, impairing dendritic cell maturation while activating survival pathways in premalignant epithelial cells (74, 75), and its induction of MMP-9 supports extracellular matrix degradation and metastasis (76, 77).

### Microbial metabolites in tumor progression

Microbial metabolites critically influence cervical tumor biology. SCFAs, generated by bacterial fermentation, show context-dependent effects: butyrate suppresses proliferation and induces apoptosis in HeLa and Caski cells (78, 79), while other studies suggest neutral or pro-tumorigenic outcomes, including Treg expansion and inflammation (80, 81). Similarly, lactic acid, protective under normal vaginal homeostasis, enhances proliferation, adhesion, and migration of cervical cancer cells, particularly via the L-enantiomer (82–84). In contrast, D-lactate has been reported to inhibit proliferation and bolster antimicrobial defense (85). Biogenic amines, found at higher levels in HPV-positive patients, exacerbate oxidative stress and DNA damage, further contributing to malignant transformation (30, 86).

### Synergistic interactions between HPV and the microbiome

Persistent infection with oncogenic HPV remains the primary etiological driver of cervical cancer (87). Dysbiosis amplifies oxidative stress, compromises epithelial barrier integrity, and impairs viral clearance, creating a microenvironment that may favor HPV genome integration (88, 89). The composition of vaginal microbiome CSTs shapes oncogenic risk in a context-dependent manner. CST I and II (dominated by *Lactobacillus crispatus* and *L. gasseri*) are, in many cohorts, associated with reduced inflammatory signaling and higher rates of HPV clearance, whereas CST III (*L. iners*) and CST IV (anaerobe-enriched communities) are more frequently linked to immune dysregulation and increased susceptibility to persistence, partly related to inerolysin activity; however, risk appears to track more consistently with functional outputs than with CST classification alone (90–92). Inflammatory signaling further amplifies HPV oncogenicity by upregulating E6/E7 expression and suppressing type I interferon responses (93, 94). Rather than representing a strictly linear cascade, current evidence supports partially overlapping and context-dependent processes. HPV persistence, dysbiosis-associated inflammation, and local immune modulation may operate in parallel, with their relative contribution varying according to host susceptibility and inflammatory tone. Although experimental systems demonstrate discrete mechanisms such as NF-κB activation and interferon suppression, the temporal hierarchy and interdependence of these processes in human cervical carcinogenesis remain incompletely defined (9, 61). Rather than discrete protective or pathogenic entities, CSTs represent a functional continuum in which microbial metabolism, epithelial integrity, and host immune tone collectively shape HPV persistence and oncogenic risk.

## The microbiome-metabolome-epigenome axis in cervical cancer progression

### Microbial dysbiosis and metabolite alterations

Cervicovaginal dysbiosis, characterized by the expansion of pathogenic taxa, has been consistently associated with metabolic reprogramming patterns linked to increased oncogenic risk (95). A key example is prostaglandin E2 (PGE2), whose elevated production correlates with *Porphyromonas* enrichment. Acting through EP2/EP4 receptor signaling, PGE2 triggers NF-κB activation in myeloid cells, thereby fueling local inflammation, while simultaneously promoting immunosuppression through the induction of regulatory dendritic cells (mregDCs) and regulatory T cells in the tumor microenvironment (96). Complementary metabolomic profiling using ultra-high-performance LC–MS/MS revealed strong associations between *Sneathia amnii* and *Lactobacillus iners* with metabolites such as 9,10-DiHOME and α-linolenic acid, compounds implicated in oxidative stress and oncogenic modulation (97). These metabolomic associations are derived from cross-sectional profiling and do not establish temporal directionality. Whether microbial shifts precede metabolite alterations or reflect secondary responses to epithelial transformation requires longitudinal confirmation (98). Similarly, GC–MS analysis of cervicovaginal fluid from HR-HPV positive women revealed lesion-specific metabolic signatures, with increased succinic acid and glucose-6-phosphate correlating with microbial shifts in *Gardnerella*, *Flavobacterium*, and *Lactobacillus* populations (99).

### Microbial metabolites, DNA methylation, and gene expression

Epigenetic regulation, including DNA methylation and histone modification, represents a pivotal mechanism in cervical carcinogenesis (100). Microbiota-derived SCFAs such as acetate, propionate, and butyrate act as potent epigenetic modulators (101, 102). By inhibiting histone deacetylases (HDACs), SCFAs modulate transcription of genes involved in inflammation and proliferation (103). Functional studies in HeLa cells show divergent effects: butyrate enhances NF-κB activation, whereas propionate exerts a suppressive influence (104). In parallel, persistent HR-HPV infection drives progressive DNA methylation, beginning in precancerous lesions and intensifying with disease progression (105). Silencing of hsa-miR-203 through hypermethylation enhances proliferation and invasion (106), while bacteria-induced methylation patterns correlate with CD8 + T cell depletion, facilitating immune evasion (107). While HDAC inhibition by short-chain fatty acids is mechanistically established at the biochemical level, associations between specific cervicovaginal taxa and host DNA methylation patterns in clinical cohorts are largely derived from correlative analyses. Functional validation directly linking defined microbial species to specific methylation events *in vivo* remains limited (108, 109).

### Epigenetic reversal via microbiome modulation

Evidence suggests that restoring a *Lactobacillus*-dominated microbiota may counteract oncogenic pathways and reinstate anti-tumor immunity. Experimental interventions with vaginal or oral *Lactobacillus* strains reduced infection burden and downregulated oncogene expression (89, 110, 111). From an epigenetic perspective, *Lactobacillus* communities are linked to enhanced expression of tumor-suppressive microRNAs such as miR-193b, which modulates genes involved in proliferation and metastasis (112). Moreover, histone lactylation, driven by microbiota-derived lactate, has been proposed as a mechanistic bridge between microbial metabolism and host epigenetic regulation (113).

### Preclinical and translational insights

In cervical organoids and mouse models, Myeong et al. (114) demonstrated that D-lactic acid from *Lactobacillus* spp. suppressed PI3K-AKT and YAP1 signaling in cervical stem cells, whereas L-lactic acid conferred therapeutic resistance. Clinically, probiotic restoration with *Lactobacillus rhamnosus* and *Lactobacillus crispatus* has been associated with improved HPV clearance and cytological outcomes (115). Although a phase I trial using *L. rhamnosus* GR-1 and *L. reuteri* RC-14 did not significantly accelerate HPV clearance, it yielded measurable improvements in cervical cytology after 6 months (116). These findings highlight the translational potential of microbiome modulation as an adjunctive approach for cervical cancer prevention and therapy.

Multi-omic profiling and experimental models converge on functional links between microbial metabolism, host epigenetic regulation, and HPV-driven carcinogenesis; however, this framework remains hypothesis-generating in humans.

## Clinical implications and microbiome-based diagnostics

Several cross-sectional and metagenomic studies identify microbiome-derived signatures as potential non-invasive biomarkers for early detection and risk stratification. Distinct bacterial taxa, microbial genetic variants, and metabolite patterns have been shown to correlate with the continuum from HPV infection to CIN and invasive carcinoma. For example, a cross-sectional 16S rRNA V3 analysis of vaginal swabs reported positive associations of *Lactobacillus iners*, *Streptococcus agalactiae*, and *Streptococcus anginosus* with increasing lesion severity (117). Similarly, *L. iners*, *Prevotella bivia*, and other commensals were linked to enhanced HPV acquisition, persistence, and progression to CIN in integrated microbial-metabolomic studies (118). Elevated abundance of *Sneathia sanguinegens*, *Anaerococcus tetradius*, and *Peptostreptococcus anaerobius*, alongside depletion of *L. jensenii*, has also been consistently observed in high-grade CIN and cervical cancer. Notably, *Sneathia* species appear as robust metagenomic markers enriched across all carcinogenic stages (119–121).

Whole-genome metagenomic sequencing has further refined the diagnostic potential of microbial signatures. In one cohort, women with cervical lesions displayed reduced *Lactobacillus* spp., while *Bifidobacteriaceae breve*, *Atopobium vaginae*, and low-abundance *L. crispatus* were associated with higher risk of preinvasive lesions and cancer. The same study identified distinct single nucleotide variation (SNV) patterns that differentiated HPV infection, preinvasive lesions, and invasive carcinoma, with SNV-based biomarkers outperforming species-level markers (AUC = 0.87 vs. 0.78) (122).

Integration of microbiome data with immune and metabolic mediators adds further prognostic granularity. A cross-sectional study demonstrated that proinflammatory cytokines (IL-6, TNFα, TRAIL), apoptotic mediators (sFas, sFasL), and angiogenic factors (FGF2, HGF, VEGF) correlated positively with cervical cancer but inversely with low-diversity vaginal microbiota. Vaginal pH and H₂O₂, key modulators of microbial beta diversity, were also associated with dysplasia and cancer progression. Multi-omic integration identified three patient clusters, cancer-associated, high-diversity/inflammation, and low-diversity/inflammation, highlighting distinct molecular trajectories of cervical carcinogenesis (123, 124). Importantly, biomarker performance frequently declines when models trained in one population are applied to geographically or ethnically distinct cohorts. Baseline microbiome structure, host genetic background, and environmental exposures modify both interaction strength and predictive accuracy. External validation across diverse settings remains essential before clinical implementation (125, 126).

Metabolomic profiling strengthens this diagnostic framework. Lesion progression has been associated with enrichment of *Atopobium* and *Sneathia*, reduction in *Lactobacillus* and *Streptococcus*, and accumulation of metabolites such as N-methylalanine, phenylacetaldehyde, succinic acid, and glucose-6-phosphate (99). Additional studies have identified ceramides and sphingomyelins as hallmarks of high-grade lesions (127) and discriminative metabolites such as 3-hydroxybutyrate, eicosenoate, and oleate-vaccenate, which distinguish cervical cancer from healthy controls with high accuracy (AUC = 0.90) (128).

Machine-learning approaches add predictive power. A random forest model identified 33 optimal bacterial predictors, with *L. iners* emerging as the most discriminative taxon (AUC = 0.952) (117). These findings are supported by a systematic review (129) and multiple cross-sectional analyses (118), which consistently associate *L. iners* with CIN2/3 risk. Furthermore, SNV-based analyses highlight specific mutational patterns (e.g., higher C > A, C > G, and T > G substitutions in preinvasive lesions vs. distinct profiles in carcinoma), reinforcing their utility for malignant risk stratification (122).

Despite strong evidence, several barriers limit clinical translation. The cervicovaginal microbiome is dynamic, influenced by hormones, diet, hygiene, and lifestyle, while geographic and demographic variability complicates biomarker generalization (121, 130). Current sequencing and analytic platforms lack sufficient sensitivity, and reproducibility is often compromised by sampling and bioinformatic variability (124). Current research focuses on (i) validating biomarkers across diverse cohorts, (ii) elucidating gut-cervical metabolic crosstalk, (iii) spatially mapping microbial intratumoral niches associated with immune escape and therapy resistance, and (iv) developing standardized, cost-effective pipelines suitable for clinical implementation, emphasizing cross-compartment, function-oriented analyses that integrate metabolic and immune readouts under rigorous contamination control to capture axis-level microbiome effects relevant to cervical cancer (131–133).

## Emerging microbiome-targeted therapeutic strategies

Probiotic interventions designed to restore microbial equilibrium are gaining increasing recognition as potential adjunctive measures in cervical cancer prevention. By re-establishing a *Lactobacillus*-dominated vaginal environment, such approaches may facilitate HPV clearance and counteract pro-tumorigenic dysbiosis. In a prospective pilot study, Verhoeven et al. reported that oral probiotic supplementation significantly enhanced HPV clearance rates (60% vs. 31% in controls) (134). Similarly, a randomized clinical trial using intravaginal *Lactobacillus rhamnosus* BMX54 documented HPV elimination in 31.2% of participants (135), while a recent pilot study employing intravaginal *Lactobacillus crispatus* demonstrated a notable reduction in viral load (136). Beyond viral clearance, preclinical models suggest that Lactobacilli possess direct antitumoral properties, including the induction of apoptosis, suppression of HPV E6/E7 oncogenes, and inhibition of pro-inflammatory pathways (137, 138). Nevertheless, the absence of large, randomized trials and safety concerns in immunocompromised women undergoing cancer therapy currently preclude their routine clinical application.

Ecological resistance may partially explain inconsistent engraftment of exogenous probiotics in dysbiotic cervicovaginal ecosystems. Established anaerobic consortia occupy metabolic niches and form biofilm-protected networks that resist recolonization by beneficial *Lactobacillus* strains. Effective therapeutic strategies must therefore consider disruption of existing interspecies cooperation and niche occupancy, not just supplementation, to shift community structure toward health-associated states. Integrating ecological principles into therapeutic design could improve prognostic power and intervention success in preventing HPV progression (139, 140).

Complementary findings from phase 2 and 3 clinical trials reinforce the translational potential of intravaginal probiotic and antimicrobial formulations. Intravaginal administration of *Lactobacillus crispatus* (Lactin-V) demonstrated significant protection against recurrent urogenital infections by maintaining high-level vaginal colonization and reducing relapse risk (141). Likewise, novel boric-acid–based antibiofilm agents, such as TOL-463, achieved high cure rates in bacterial vaginosis and vulvovaginal candidiasis while preserving protective *Lactobacillus* populations (142). A single-dose oral formulation of secnidazole further proved to be well-tolerated and effective in eradicating biofilm-associated anaerobes without compromising beneficial microbiota (143). Collectively, these data suggest that vaginal biofilm disruption and targeted microbial repletion can be safely combined to restore mucosal homeostasis and potentially enhance therapeutic outcomes in HPV-related neoplasia.

Parallel evidence highlights the potential of microbiome-modulating strategies to augment immunotherapy efficacy and mitigate treatment-related toxicities in oncology. A recent meta-analysis encompassing retrospective and prospective studies demonstrated that probiotics significantly improved both overall and progression-free survival in patients with non-small cell lung cancer, melanoma, and other solid tumors receiving immune checkpoint inhibitors (ICIs), particularly in antibiotic-exposed individuals (144, 145). Mechanistic data indicate that antibiotic exposure reduces the antitumor efficacy of anti-PD-L1 therapy in non-small cell lung cancer patients with high PD-L1 expression (146). Although ICIs are not yet standard in cervical cancer therapy, multiple ongoing clinical trials are assessing pembrolizumab in newly diagnosed or recurrent/metastatic disease (147). Emerging translational frameworks propose combining probiotic consortia or prebiotic substrates with ICIs to reshape systemic immunity and enhance checkpoint responsiveness (120). These trials underscore the possibility that microbiome modulation could enhance ICI responsiveness in cervical cancer as well.

Beyond probiotics, additional strategies aim to selectively target pro-tumorigenic microbial niches while sparing protective commensals. These include localized antimicrobials, engineered probiotics, and evidence-based prebiotic or dietary interventions, contingent on the identification of microbial “hotspots” that drive carcinogenic pathways (133). Prebiotic-based therapies are also emerging; for instance, Papilocare, a topical gel enriched with the prebiotic Bioecolia, has demonstrated efficacy in HPV-related cervical lesions, though further validation is warranted (148). Another promising frontier is microbiome editing, in which engineered microorganisms or derivatives such as bacterially derived antibodies are designed to selectively eliminate tumor cells (149). Current and experimental protocols also encompass biofilm disruptors, vaginally delivered *Lactobacillus* formulations, and next-generation antimicrobials that integrate precision delivery systems with host-compatible substrates (142, 143). While conceptually compelling, these approaches remain experimental and require rigorous translational evaluation before clinical adoption.

Vaginal microbiota transplantation (VMT) represents another innovative intervention, involving the transfer of vaginal fluid from healthy donors to individuals with vaginal dysbiosis. Early studies suggest that VMT can reconstitute a balanced microbial community (120), and an ongoing Phase I-II clinical trial is assessing its safety and efficacy (NCT04046900). Although VMT may theoretically shift the vaginal microbiota from an inflammatory to a protective state in HPV-positive or lesion-bearing patients, the approach remains investigational, with unresolved issues including donor screening, standardization of protocols, and potential risks such as pathogen transmission or long-term immune consequences (150). Recent reviews emphasize that the future of VMT will depend on integration with precision-profiling frameworks, where donor selection is guided by genomic and metabolomic compatibility (120).

Antibiotic interventions, though capable of altering both gut and vaginal microbiota composition, risk destabilizing protective microbial communities. Antibiotic treatment has been shown to reduce gut microbiota diversity, which, due to the interconnection between gut and vaginal microbiomes, may also disrupt vaginal homeostasis (151). The combined use of antibiotics and probiotics has thus been proposed as a way to modulate both ecosystems and potentially influence cervical lesion dynamics, though this remains experimental. Current guidance from the European Society of Gynaecological Oncology emphasizes the urgent need for mechanistic studies, while cautioning that available evidence does not yet support routine microbiota manipulation for cervical cancer prevention or therapy (152).

Importantly, probiotics may alleviate treatment-related complications such as diarrhea, oral mucositis, and infections, as shown in umbrella reviews of interventional trials (153). Yet, their use in immunocompromised populations remains tightly regulated by health authorities including the FDA, FAO/WHO, NMPA, ANVISA, and FSANZ. Advances in shotgun metagenomics have revealed functional alterations in the vaginal microbiome linked to persistent HPV infection and cervical dysplasia (154). Still, the clinical utility of microbiota modulation for preventing or regressing cervical lesions has not been established, and interactions with cofactors such as smoking, co-infections, hormonal contraception, and nutritional status must be carefully considered (155).

Microbiome-based interventions, including probiotics, prebiotics, microbiome editing, and vaginal microbiota transplantation, constitute a rapidly advancing field with strong translational potential in cervical cancer. However, regulatory barriers, limited mechanistic evidence, and unresolved safety concerns currently restrict their use to clinical research settings. Until large-scale data become available, implementation should remain within rigorously controlled clinical trials.

## Conclusion and future perspectives

Current data indicate that the microbiome acts as a dynamic and context-dependent contributor that modulates carcinogenic trajectories rather than functioning as a standalone etiological factor. Studies have consistently shown that cervicovaginal dysbiosis, marked by depletion of *Lactobacillus* species and enrichment of anaerobic taxa such as *Gardnerella*, *Atopobium*, and *Sneathia*, promotes persistent HPV infection and progression to CIN and invasive carcinoma (89, 121, 152). These microbial alterations have been individually associated with chronic inflammation, oxidative stress, epithelial barrier disruption, and modulation of host immune responses. However, the sequencing, interaction strength, and causal hierarchy among these processes remain to be fully clarified (22, 30, 97). Crucially, cervicovaginal microbiome states should not be interpreted as binary healthy or pathogenic configurations. Protective or deleterious effects emerge from context-dependent interactions among microbial composition, metabolic activity, host immunity, hormonal milieu, and population-specific factors, rather than from *Lactobacillus* dominance alone (137, 156). Cervicovaginal microbiome configurations therefore shape risk in a probabilistic manner. Their biological impact depends on interaction strength, temporal stability, and host context, rather than on fixed taxonomic dominance alone (157–159).

Beyond descriptive associations, recent research has advanced toward mechanistic and translational understanding. The role of bacterial metabolites, particularly SCFAs such as butyrate, has gained prominence. Butyrate exerts tumor-suppressive properties by inhibiting cervical cancer cell proliferation, inducing apoptosis, and reshaping epigenetic regulation, positioning it as a metabolite of therapeutic interest (79). Likewise, microbiome-metabolome integration studies reveal correlations between cervicovaginal microbial profiles and altered host metabolites such as 9,10-DiHOME and glycocholic acid, which may serve as biomarkers for HPV persistence and early detection of cervical lesions. Circadian misalignment has also been linked to shifts in microbial taxa, including *Lactobacillus* and *Bacteroides*, whose rhythmic activity modulates epithelial defense and inflammatory tone, adding a temporal dimension to cervical carcinogenesis (30, 160). Leveraging microbiome-derived biomarkers enables more precise diagnostics and risk stratification.

Therapeutically, the modulation of the cervicovaginal microbiota emerges as a promising strategy. Probiotic and prebiotic interventions have demonstrated potential to restore *Lactobacillus* dominance, enhance HPV clearance, and reduce recurrence of cervical lesions (137, 152). Probiotic formulations such as oral and intravaginal *Lactobacillus rhamnosus* and *Lactobacillus crispatus* have increased HPV clearance and decreased viral load in clinical trials (134). These bacteria exhibit antitumoral activity by suppressing HPV E6/E7 oncogenes, inducing apoptosis, and reducing pro-inflammatory signaling (138). Complementary studies with *Lactobacillus crispatus* (Lactin-V) and antibiofilm agents like TOL-463 further demonstrate that microbial repletion coupled with biofilm disruption can safely restore vaginal homeostasis (141).

Emerging evidence also links microbiome modulation with improved cancer immunotherapy outcomes. Probiotics enhance survival in patients treated with immune checkpoint inhibitors (ICIs), mitigating the negative impact of antibiotics on anti–PD-L1 efficacy (145). Although ICIs are not yet standard in cervical cancer, ongoing pembrolizumab trials suggest potential synergy when combined with microbiome-based interventions (147). Other novel strategies, including prebiotic gels such as Papilocare, microbiome editing with engineered probiotics, and vaginal microbiota transplantation (VMT), offer innovative approaches to restore protective microbial communities (148). However, rigorous donor screening, long-term monitoring, and ethical frameworks are required before these therapies reach clinical application (120).

The intratumoral microbiome also represents an emerging dimension of investigation. Evidence indicates that microbial communities within cervical tumors may influence oncogene expression, immune evasion, and therapeutic response (89). Integration of intratumoral and cervicovaginal microbiome profiles could uncover novel microbial signatures predictive of prognosis and therapeutic outcomes. Additionally, cross-talk between the gut and genital tract microbiota has been recognized as a key axis in modulating estrogen metabolism, immune responses, and systemic inflammation, further linking distant microbial ecosystems to cervical cancer progression (22, 156, 161). Deciphering these interactions could inspire multi-level interventions targeting both local and systemic microbial networks.

From a clinical standpoint, the potential of microbiome-based diagnostics and therapeutics aligns with the broader agenda of precision oncology. Predictive models built on microbiome profiles demonstrate high discriminatory power for distinguishing healthy, HPV-infected, CIN, and invasive cancer states (22, 79, 120). These tools, if validated in multi-ethnic and geographically diverse cohorts, could significantly improve screening programs, particularly in low-resource settings where cervical cancer burden remains disproportionately high (30, 137). Embedding microbiome markers within existing HPV screening frameworks may refine triage, reduce overtreatment, and optimize resource allocation, especially where vaccination remains suboptimal.

Looking ahead, several challenges and opportunities shape future research. Methodological standardization is essential, as heterogeneity in sampling, sequencing, and data analysis hinders comparability among studies (22, 97). Longitudinal designs and harmonized protocols are needed to capture dynamic microbiome shifts during disease progression and therapy. Multi-omics integration, encompassing metagenomics, metabolomics, transcriptomics, and immunogenomics, will be key to disentangling host-microbe-metabolite networks underlying cervical oncogenesis (30, 79). Beyond technical considerations, the clinical translation of microbiome-based interventions raises important ethical and regulatory challenges. Robust governance frameworks will be required to ensure the safety, efficacy, and long-term monitoring of emerging strategies such as FMT and rationally engineered microbial consortia, while also addressing issues of standardization, patient stratification, and equitable access across healthcare systems (120, 162).
